# Development of an *in vitro *cleavage assay system to examine vaccinia virus I7L cysteine proteinase activity

**DOI:** 10.1186/1743-422X-2-63

**Published:** 2005-08-16

**Authors:** Chelsea M Byrd, Dennis E Hruby

**Affiliations:** 1 Molecular and Cellular Biology Program, Oregon State University, 220 Nash Hall, Corvallis, Oregon, 97331, USA; 2 Siga Technologies, 4575 SW Research Way, Suite 230, Corvallis, Oregon, 97333, USA

## Abstract

Through the use of transient expression assays and directed genetics, the vaccinia virus (VV) I7L gene product has been implicated as the major maturational proteinase required for viral core protein cleavage to occur during virion assembly. To confirm this hypothesis and to enable a biochemical examination of the I7L cysteine proteinase, an *in vitro *cleavage assay was developed. Using extracts of VV infected cells as the source of enzyme, reaction conditions were developed which allowed accurate and efficient cleavage of exogenously added core protein precursors (P4a, P4b and P25K). The cleavage reaction proceeded in a time-dependent manner and was optimal when incubated at 25°C. I7L-mediated cleavage was not affected by selected inhibitors of metalloproteinases, aspartic acid proteinases or serine proteinases (EDTA, pepstatin, and PMSF, respectively), but was sensitive to several general cysteine proteinase inhibitors (E-64, EST, Iodoacetic acid, and NEM) as well as the I7L active site inhibitor TTP-6171 [C. Byrd *et al*., J. Virol. 78:12147–12156 (2004)]. Finally, in antibody pull down experiments, it could be demonstrated that monospecific αI7L serum depleted the enzyme activity whereas control sera including αG1L, directed against the VV metalloproteinase, did not. Taken together, these data provide biochemical evidence that I7L is a cysteine proteinase which is directly involved in VV core protein cleavage. Furthermore, establishment of this I7L-mediated *in vitro *cleavage assay should enable future studies into the enzymology and co-factor requirements of the proteolysis reaction, and facilitate antiviral drug development against this essential target.

## Background

The *Orthopoxviridae *include vaccinia virus, camelpox, cowpox, ectromelia, monkeypox, raccoonpox, skunkpox, taterapox, volepox, and variola. Viruses in this family are the cause of numerous diseases including smallpox (variola), and recent human outbreaks of monkeypox. Orthopoxviruses are large double-stranded DNA viruses that are unique amongst DNA viruses in that they replicate exclusively within the cytoplasm of infected cells. Vaccinia virus (VV) is the most extensively studied virus in this group and is the prototypic member. The genome of VV is predicted to encode over 200 open reading frames. VV expresses its genetic information in three stages, as early, intermediate, and late genes. The early genes, which account for approximately half of the genome and are transcribed prior to DNA replication, encode many of the proteins involved in viral DNA replication and intermediate gene expression. The intermediate genes, of which only a handful have been identified, are expressed after the onset of DNA replication, and encode proteins that are activators of late gene expression. The late genes encode many proteins required for the transcription of early genes, the viral structural proteins and the enzymes necessary to process these proteins into their mature form.

Many viruses use proteolytic processing as a key step in their developmental cycle. RNA viruses and retroviruses commonly undergo formative proteolysis in which large polyproteins are cleaved by viral encoded proteinases to produce the structural and nonstructural proteins required for morphogenesis. DNA viruses such as poxviruses and adenoviruses commonly use another type of proteolysis, called morphogenic proteolysis where precursor proteins are first synthesized and then cleaved by viral proteinases to produce the mature form of the protein. The mature protein then plays an essential role in virion formation. During VV assembly, as the spherical immature virions (IVs) are maturing into the first infectious form of vaccinia virus, intracellular mature virus (IMV), a series of events takes place including proteolytic processing of viral core proteins [1-4].

Our laboratory has worked to identify and characterize the proteinases of VV in order to understand their regulation, function, and biochemistry, with a long term goal of developing inhibitors of these enzymes as antiviral drugs. The gene product of the I7L open reading frame recently has been suggested to be the core protein proteinase of VV through the use of an *in vivo trans *processing assay [5,6]. I7L is an essential late gene, as shown through temperature sensitive mutant viruses [7,8] and conditional lethal mutant viruses [9,10] where under non-permissive conditions, viral morphogenesis is blocked prior to the formation of IMV. I7L is predicted to be a 47 kDa cysteine proteinase that cleaves the major core protein precursors P4a, P4b, and P25K, products of the A10L, A3L, and L4R open reading frames respectively, at a novel Ala-Gly-Xaa cleavage site with cleavage occurring after the glycine residue [5,6]. I7L also is likely to be responsible for cleavage of the A17 membrane protein, at an Ala-Gly-Ala site [9]. This consensus Ala-Gly-Xaa cleavage site of vaccinia is similar to that used for both the adenovirus and African swine fever virus proteinases which cleave after the second glycine in a Gly-Gly-Xaa motif [11,12].

Comparative sequence analysis has suggested that the VV I7L proteinase is related to the ASFV and adenovirus cysteine proteinases and may form a new family of SUMO-1 related enzymes [13,12]. The nucleophilic cysteine is responsible for cleavage and is activated by the imidazol group of the catalytic histidine residue. Substrate specificity is determined by the substrate binding pocket and is unique for each proteinase. Several critical residues have been identified as being necessary for enzymatic activity of I7L including the catalytic triad residues [6]. Based on the identification of the catalytic residues and the predicted structure of the I7L proteinase, a new class of small molecule inhibitors was developed that are capable of inhibiting the replication of VV, and were found to specifically target I7L through the generation of drug resistant mutant viruses with the mutations mapping to I7L [14].

To date, direct studies on the enzymology of I7L-mediated proteolysis have not been possible due to the absence of a suitable biochemical assay. In the experiments reported here, we describe the development of an *in vitro *I7L-mediated cleavage assay. We have used this system to obtain both biochemical and immunological data to prove that I7L is directly involved in cleavage of the major VV core protein precursors. Having this assay available will now facilitate biochemistry of the I7L enzyme and identification of all the required reaction components to be undertaken.

## Results

To date, all studies of VV I7L activity have been carried out indirectly in transfected/infected tissue culture cells. Although this approach has provided some important insights into I7L biology, it is limited with respect to the study of I7L enzymology and identification of all the *cis *and *trans *factors required for substrate identification and catalysis. In order to approach these questions, we have sought to develop an *in vitro *cleavage assay for I7L. Thus far, the obvious approaches of expressing and purifying I7L from prokaryotic and eukaryotic expression vectors and combining with peptides or proteins containing a canonical A-G-X cleavage site have not been successful (data not shown), perhaps due to either the lack of essential co-factors or inappropriate assay conditions. As an alternative approach, we sought to develop a cleavage assay using infected cell extracts as the source of I7L activity and labeled core protein precursors made *in vitro *as the substrate. If successful, this system would provide the starting point for a dissection of the essential reaction components.

### In vitro Processing of Core Protein Precursors

**Figure 1:**
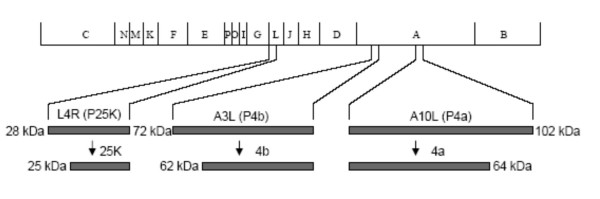
**Schematic representation of the major core protein precursor cleavage products**. The vaccinia virus genome is represented depicting three of the major core protein precursors, the gene products of the L4R, A10L, and A3L open reading frames, P25K, P4a, and P4b respectively. The precursors are shown being cleaved into their mature form. Molecular mass is indicated.

**Figure 2:**
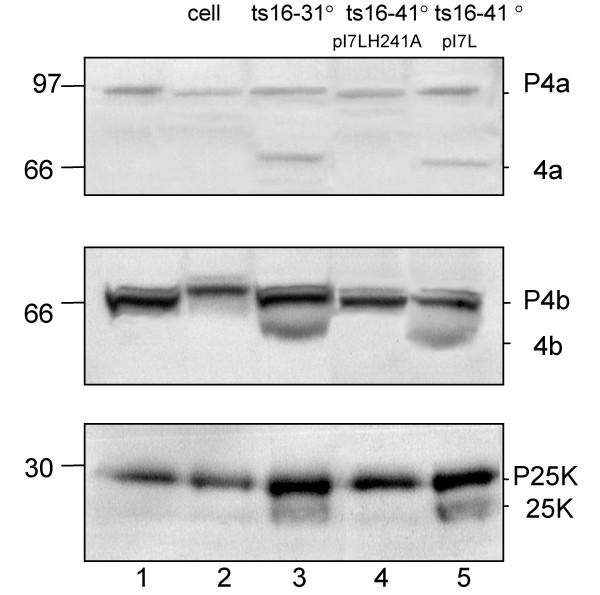
***In vitro *proteolytic processing of P4a, P4b, and P25K**. 1 μl of TNT produced substrate either P4a (A), P4b (B), or P25K (C) was mixed with 5 μl of Hepes buffer and 14 μl of enzyme extracts, either from uninfected cells, or cells infected with ts16 at the permissive or non-permissive temperature. At the non-permissive temperature, plasmid borne I7L, either wild-type (pI7L) or mutant I7L (pI7LH241A) was transfected in as the source of enzyme. The reaction was incubated at 29°C for 3 hrs before being stopped by the addition of SDS sample buffer. Molecular weight is indicated on the left and the core protein precursor and product on the right. Lane 1 is substrate alone, lane 2 is substrate mixed with cellular extracts and lanes 3–5 are substrate mixed with the enzyme extract indicated.

The three major core protein precursors P4a (A10L), P4b (A3L), and P25K (L4R) which are known to be cleaved to a mature form (Figure 1) were cloned into plasmid vectors driven off of a T7 promoter to be used as a source of substrate for the assay. To investigate the ability of I7L to cleave the P4a, P4b, and P25K substrates *in vitro*, we have used a system where the substrates are produced from an *in vitro *transcription and translation assay using rabbit reticulocyte lysates and then mixed with I7L expressed from virus infected cells. BSC40 cells are infected with *ts16*, a temperature sensitive mutant virus in which the responsible mutation maps to I7L. The virus infected cells are incubated at the non-permissive temperature and transfected with plasmids expressing either wild-type I7L (pI7L) or I7L with the catalytic histidine residue mutated to an alanine (pI7LH241A). The extracts are prepared as described in the Materials and Methods. The extracts are mixed and incubated with the substrates for 3 hrs and then analyzed through SDS-PAGE and chemiluminescent detection. As shown in Figure 2, a specific band corresponding to unprocessed P4a (top panel), P4b (middle panel), or P25K (bottom panel) is produced when the substrate is run alone. When mixed with cellular extracts, or extracts from cells infected with *ts16 *at the non-permissive temperature and transfected with mutant I7L, no cleavage products are observed. However, when mixed with extracts from either cells infected with *ts16 *at the permissive temperature or cells infected with *ts16 *at the non-permissive temperature transfected with wild-type I7L, the cleaved products 4a, 4b, and 25K are observed. Substrates with mutated A-G-X sites were not cleaved indicating that cleavage was occurring at the correct sites (data not shown). For the rest of the reported studies, P25K was used as the source of substrate since it gave the best cleavage profile.

### Processing Kinetics of Core Protein Precursors

**Figure 3:**
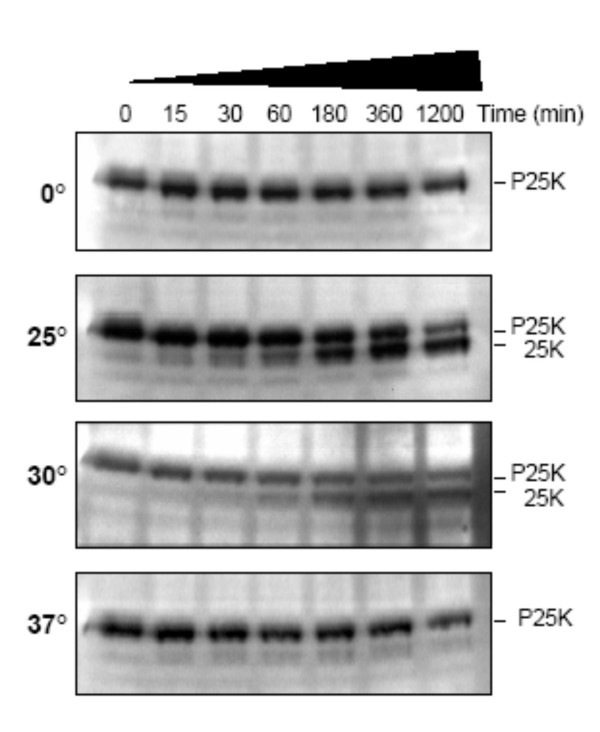
**Processing kinetics of P25K**. Samples were incubated at either 0°C (A), 25°C (B), 30°C (C), or 37°C (D) for up to 20 hrs, harvested at the indicated times and the reaction stopped by the addition of SDS sample buffer. Incubation temperature is indicated on the left and P25K precursor and 25K mature product are indicated on the right.

To determine the optimal temperature and kinetics of processing of the core protein precursors in the *in vitro *cleavage assay, a time course of I7L-mediated processing at various temperatures was performed. As shown in Figure 3A, at 0°C, no processing was observed during the 20 hr time period. At 25°C, a gradual increase in the amount of P25K cleavage product was observed starting at 15 min and increasing throughout the 20 hr incubation period (Fig. 3B). Compared with the rate of cleavage at 25°C, cleavage was slower at 30°C (Fig. 3C), starting around 30 min and increasing through the 20 hr period, but never to the same level as at 25°C. Processing is greatly reduced at 37°C with only a faint processed band ever appearing (Fig 3D).

### Influence of Thiol Reagents on the Protease Activity

Based on its sequence similarity to the adenovirus protease, the African swine fever virus protease, and an ubiquitin-degrading enzyme in yeast, as well as the identity of a catalytic triad composed of histidine, cysteine, and aspartic acid, I7L has been classified as a cysteine proteinase. The thiol reagents dithiothreitol (DTT) and cysteine have been shown to enhance the cleavage activity of the adenovirus protease in an *in vitro *peptide cleavage assay [15]. To determine whether these agents have a similar effect on the activity of I7L, they were added to the *in vitro *assay in a final concentration from 0–10 mM. However, no increase in cleavage activity was observed with the addition of either DTT or cysteine (data not shown). It is possible that once purified recombinant enzyme is produced these thiol reagents may increase its activity.

### Effect of Inhibitors on Protease Activity and Characterization as a Cysteine Proteinase

**Figure 4:**
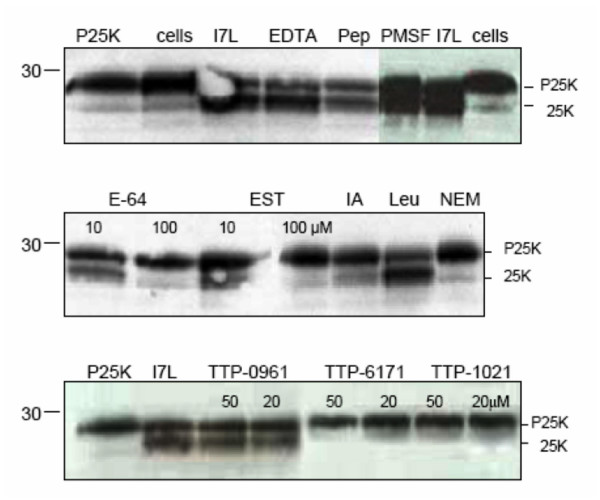
**Effect of inhibitors on *in vitro *processing**. Various concentrations of protease inhibitors were added to the *in vitro *processing assay for 6 hr at 29°C. The first lane is P25K expressed alone with no extract added. The second lane is P25K mixed with cellular extracts and the third lane is P25K mixed with I7L enzyme extracts. Each of the remaining lanes has P25K mixed with I7L enzyme extracts plus indicated inhibitor. Ethylenediaminetetraacetic acid (EDTA) was used at 1 mM. Pepstatin A, Pep, was used at 10 μM. Phenlymethanesulfonyl fluoride (PMSF) was used at 1 mM. N-(trans-Epoxysuccinyl)-L-leucine 4-guanidinobutylamide *trans*-Epoxysuccinyl-L-leucylamido(4-guanidino)butane (E-64) and a related product EST, were both used at 10 μM and 100 μM concentrations. Iodoacetic acid (IA) was used at 1 mM. Leupeptin (Leu) was used at 1 mM, and N-ethlymaleimide (NEM) was used at 2.5 mM. The concentrations of TTP-6171, TTP-1021, and TTP-0961 are indicated. The table indicates the concentration of inhibitor used and whether cleavage activity was observed.

The *in vitro *assay allowed us to test the effects of various protease inhibitors, as well as specific small molecule inhibitors on the activity of I7L. As shown in Figure 4 and Table 1, the metalloproteinase inhibitor ethylenediaminetetraacetic acid (EDTA), the aspartic proteinase inhibitor pepstatin, and the serine proteinase inhibitor phenylmethanesulfonyl (PMSF) had no detectable effect on cleavage activity. The cysteine proteinase inhibitors iodoacetic acid (IA) and *N*-ethylmaleimide (NEM) efficiently blocked I7L mediated proteolysis of P25K. The cysteine proteinase inhibitors E-64 and EST were shown to inhibit protease activity at a relatively high concentration, but not at the lower concentration tested. This is consistent with what has been observed for both the adenovirus protease [16], and the African swine fever virus protease [17]. The failure of E-64 to inhibit protease activity at the lower concentration tested, and the location of the active site residues may suggest that each of these enzymes are not conventional papain-like enzymes, but may be a new family of cysteine proteinases. The cysteine protease inhibitor leupeptin also failed to inhibit protease activity, although this lack of inhibition was also observed with the adenovirus proteinase [16].

**Table 1:** Effect of inhibitors on *in vitro *processing.

**Inhibitor**	**Name**	**Concentration**	**Inhibit Cleavage**
Metalloproteinase	EDTA	1 mM	No
Aspartic acid proteinase	Pepstatin	10 μM	No
Serine proteinase	PMSF	1 mM	No
Cysteine proteinase	E-64	10 μM	No
	E-64	100 μM	Yes
	EST	10 μM	No
	EST	100 μM	Yes
	IA	1 mM	Yes
	Leupeptin	1 mM	No
	NEM	2.5 mM	Yes
TTP inhibitors	TTP-6171	50 μM	Yes
	TTP-6171	20 μM	Yes
	TTP-1021	50 μM	Yes
	TTP-1021	20 μM	Yes
	TTP-0961	50 μM	No
	TTP-0961	20 μM	No

Next we wanted to determine if the small molecule I7L inhibitors previously developed as antiviral drug candidates [14] could be shown to specifically inhibit the activity of I7L in the *in vitro *assay. The compound TTP-6171 has been shown to inhibit viral replication in tissue culture, with drug resistant virus mutations mapping to I7L [14]. Here we see that this compound along with TTP-1021, which was also found to inhibit I7L in tissue culture, inhibits the processing of P25K *in vitro*. However the compound TTP-0961, which was not found to generate resistant mutants in the I7L gene (data not shown), does not inhibit cleavage. These results demonstrate that this assay can be used for the screening of specific I7L inhibitors and confirms that this class of molecules targets I7L.

### Effects of I7L antibody competition on cleavage

**Figure 5:**
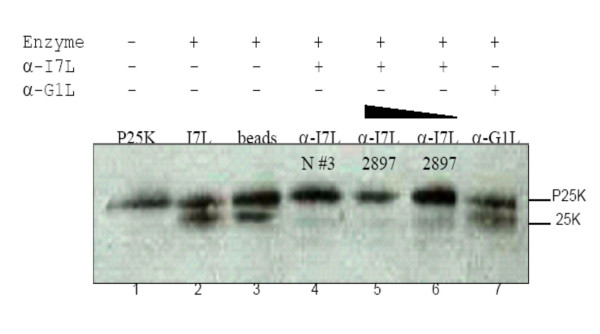
**Effect of antibody competition on *in vitro *processing**. Lane 1 is P25K expressed alone. Lane 2 is P25K mixed with I7L enzyme extracts. Lane 3 is P25K mixed with I7L extracts that have been diluted with Hepes buffer and treated with Sepharose beads. Lanes 4, 5, and 6 are P25K mixed with I7L extracts that have been incubated overnight with different I7L antiserum (indicated on each lane), treated with Sepharose beads and the antibody complex removed by centrifugation. Lane 7 is P25K mixed with I7L extracts incubated with G1L antiserum as above.

To directly demonstrate that the cleavage observed in the *in vitro *assay requires the presence of I7L, increasing concentrations of I7L specific antiserum were added to the enzyme extracts overnight, and then the complex was precipitated with Protein A sepharose beads to deplete the extract of I7L and any associated co-factors. As shown in Figure 5, both of the I7L antisera tested inhibited cleavage of P25K while an antiserum targeting a different VV gene product, G1L, did not inhibit cleavage.

## Discussion

In this report, a cell-free transcription and translation system was used to develop an *in vitro *cleavage assay for the VV cysteine proteinase I7L. Proteolytic activity was obtained by co-expression of I7L in *ts16 *infected cells at the non-permissive temperature. Each of the major core protein precursors, P4a, P4b, and P25K, were shown to be cleaved to their mature products by I7L using the *in vitro *assay. Evidence that this cleavage is specific to I7L was shown through the fact that expressing a mutant form of I7L resulted in the inability to cleave the core protein precursors. Antibody pull down experiments with αI7L supported the conclusion that I7L plays a direct role in the proteolytic reaction.

A time course of processing at various temperatures indicated that for this particular assay, the optimal temperature for the reaction to be carried out at is 25°C with processing beginning as soon as 15 minutes after addition of enzyme and increasing as time progresses. The cleavage reaction was never driven to completion and this may be due to a lack of replenishing co-factors or the enzyme may have been used up in the reaction. It was surprising that the optimal reaction temperature was 25°C instead of 37°C which is the optimal growth temperature for VV in cell culture. One possible explanation is that I7L is present at high concentrations in the extract and one can measure marginal activity at low temperature, whereas at higher temperatures other proteinases are activated which degrade the I7L enzyme.

Known cysteine protease inhibitors such as E-64, iodoacetic acid, and NEM were shown to inhibit the *in-vitro *cleavage reaction while the metalloproteinase inhibitor EDTA, the aspartic acid protease inhibitor pepstatin, and the serine protease inhibitor PMSF all failed to inhibit the cleavage reaction indicating that the enzyme responsible for cleavage is a cysteine protease. Interestingly the cysteine protease inhibitors leupeptin, and low concentrations of E-64 did not inhibit the reaction. These cysteine protease inhibitors were also not shown to be effective against either the African Swine Fever Virus protease [17] or the adenovirus protease [16], further providing support for the theory that these enzymes may form a new family of cysteine proteases that differ from papain-like cysteine proteases.

Of particular interest, the small molecule inhibitors designed to fit into the active site pocket of I7L and previously shown to inhibit viral replication [14], were found to be active in inhibiting the *in vitro *cleavage reaction described here. A related compound (TTP-0961) that was not found to map to I7L was not able to abolish cleavage. This indicated that this assay may be useful for high-throughput screening of compounds to identify those that have specific activity for I7L.

## Conclusion

Until this point, all work demonstrating that I7L is the core protein proteinase has been done through transient-expression assays and the use of conditional lethal viruses in tissue culture [9,5,6,10]. The data obtained has indicated that I7L is essential for these processing activities, it did not rule out the possibility that some other factor or enzyme was also required for this activity to occur. Through the use of an *in vitro *assay we have shown that I7L is capable of cleaving the core protein precursors but that an additional co-factor is required for this activity to occur since expression of the enzyme through cell-free translation produced inactive enzyme. The co-factor(s) necessary for cleavage have yet to be determined. However, having the assay described in this report available will now enable a reductive analysis to be conducted to identify all the essential components of the reaction and to study their individual biochemical characteristics.

## Methods

### Cells and Viruses

BSC_40 _cells [18] were grown in Eagle's minimal essential medium containing 5% fetal calf serum (FCS) (Sigma, St. Louis, MO), 2 mM glutamine (Invitrogen, Carlsbad, CA), and 15 μg/ml gentamicin sulfate (Invitrogen) in a 37°C incubator with 5% CO_2_. Purified *ts16 *Vaccinia virus was prepared as described [19]. *Escherichia coli *strains were grown in Luria-Bertani broth or on Luria-Bertani medium containing 1.5% agar and ampicillin at 50 μg/ml.

### Plasmids

The A10L (P4a) gene was amplified by polymerase chain reaction using oligonucleotides KH10 (5' CATGCCATGGATGATGCCTATTAAGTCAATAGTTACT CTT-3') and KH11 (5'-CCGCTCGAGTTATTCATCATCAAAAGAGACAGAGTC-3'), digested with NcoI and XhoI, and cloned into the pTM1 vector, yielding pTM-P4a which utilizes a T7 promoter for expression. The A3L (P4b) gene was amplified using oligonucleotides KH08 (5'-CATGCCATGGATGGAAGCCGTGGTCAATAG-3') and KH09 (5'-TCCCCCGGGCTAAAAATAGTTCTGTAATATGTCTAGCGCT-3'), digested with NcoI and SmaI, and cloned into the pTM1 vector to yield pTM-P4b. The L4R (P25K) gene was amplified using oligonucleotides DN51 (5'-CATGCCATG GATGAGTCTACTGCTAGAAAAC-3') and KH07 (5'-CCGCTCGAGTCAATCCTTT GTCG-3'), digested with NcoI and XhoI, and cloned into the pTM1 vector to yield pTM-P25K. The pI7L and pI7LH241A plasmids were described in Byrd *et al*., 2002 [5].

### Preparation of polyprotein or proteinase-containing extracts

Confluent monolayers of BSC_40 _cells in 6-well plates were infected with *ts*16 VV at a multiplicity of infection of 2 plaque-forming units per cell and transfected with 2 μg of plasmid DNA (either pI7L, or pI7LH241A) using DMRIE-C (Invitrogen) following the manufacturer's indications. Infected cells were incubated either at the permissive temperature of 31.5°C or the non-permissive temperature of 39°C. Cells were harvested at 24 h post-infection by pipetting up and down to lift the cells from the surface. The infected cells were centrifuged at 10,000 × g for 10 min, the supernatant was aspirated off, and the pellet was resuspended in 500 μL homogenization buffer containing 20 mM HEPES (pH 7.4), 0.28 M sucrose, 2 mM EDTA. This was passed through a 25-gauge syringe 15 times. The homogenate was centrifuged at 700 × g for 5 min to separate the nuclei and unbroken cells from the supernatant. The supernatant was centrifuged at 100,000 × g for 30 min at 4°C to separate the membrane/particulate material from the supernatant. The supernatant was used as the source of enzyme.

Coupled TNT reactions with T7 RNA polymerase were performed according to the manufacturer's instructions (Promega Corporation, Madison, Wisconsin) as a source of substrate. Briefly, the TNT reactions were performed at 30°C in a final volume of 25 μL with 1 μg of plasmid DNA, using the non-radioactive Transcend label (biotinylated lysine residues are incorporated in the protein) provided with the kit for detection of protein.

### In vitro cleavage assay

Reactions were performed at the indicated temperature in a final volume of 20 μL containing 1 μL of substrate, 13 μL of enzyme extract, and 6 μL of 20 mM HEPES (pH 7.4) buffer, pH 7.4. After the indicated times, the reaction was stopped by the addition of SDS sample buffer, and the samples were subjected to SDS-polyacrylamide gel electrophoresis. The results were analyzed by immunoblotting following the instructions provided by the TNT kit.

### Inhibitor studies

For inhibitor studies, the reactions described above were incubated for 6 hr in the presence or absence of the following protease inhibitors: 1 mM phenylmethanesulfonyl fluoride (PMSF) (Sigma), 10 μM Pepstatin A (Sigma), 1 mM ethylenediaminetetraacetic acid (EDTA) (Sigma), 10 μM or 100 μM N-(trans-Epoxysuccinyl)-L-leucine 4-guanidinobutylamide *trans*-Epoxysuccinyl-L-leucylamido(4-guanidino)butane (E-64) (Sigma), 1 mM iodoacetic acid (Sigma), 10 μM or 100 μM Leupeptin (Roche, Indianapolis, IN), 2.5 mM *N*-ethylmaleimide (NEM) (Sigma). For I7L specific inhibition studies, the reactions described above were incubated for 6 hr in the presence or absence of TTP-6171, TTP-1021, or TTP-0961 [14] at 5 μM or 20 μM final concentrations.

### Antibody competition studies

For the antibody competition studies, 25 μl of I7L or G1L specific antiserum was added to 25 μL of enzyme extract on a rotating shaker overnight at 4°C. ProteinA: Sepharose beads (Amersham Biosciences, Uppsla, Sweden) were added for 3 hrs and the antibody complex was centrifuged to pull down the I7L enzyme. The supernatant was used as the source of extract in the *in vitro *assay described above. As a control, enzyme extract was mixed with buffer instead of antibody and treated with beads in a similar manner.

## Competing interests

The author(s) declare that they have no competing interests.

## Authors' contributions

CMB conceived the study, conducted all the experiments and wrote the manuscript. DEH coordinated the research efforts and edited the paper. Both authors read and approved the final manuscript.
